# Giant Appendiceal Leiomyosarcoma: A Rare and Unusual Tumour

**DOI:** 10.1155/2011/384762

**Published:** 2011-09-07

**Authors:** Christine Natalia, Cherry E. Koh, Peter J. Lee

**Affiliations:** ^1^Department of Colorectal Surgery, Royal Prince Alfred Hospital, Camperdown, NSW 2050, Australia; ^2^Surgical Outcomes Research (SOuRCe), Royal Prince Alfred Hospital, Camperdown, NSW 2050, Australia; ^3^Royal Prince Alfred Hospital Medical Centre, Suite 415/ 100 Carillon Avenue, Newtown, NSW 2042, Australia

## Abstract

Appendiceal tumours are uncommon but may be present in 0.9–1.4% of all appendicectomy specimens. While carcinoid tumours and adenocarcinomas comprise the majority of appendiceal tumours, rarely, lymphomas or sarcomas may also present in the appendix. Appendiceal leiomyosarcomas are rare, and to date, only a handful of cases have been reported. The current paper presents a case of giant appendiceal leiomyosarcoma followed by a review of the literature.

## 1. Case Presentation

**Figure 1:**
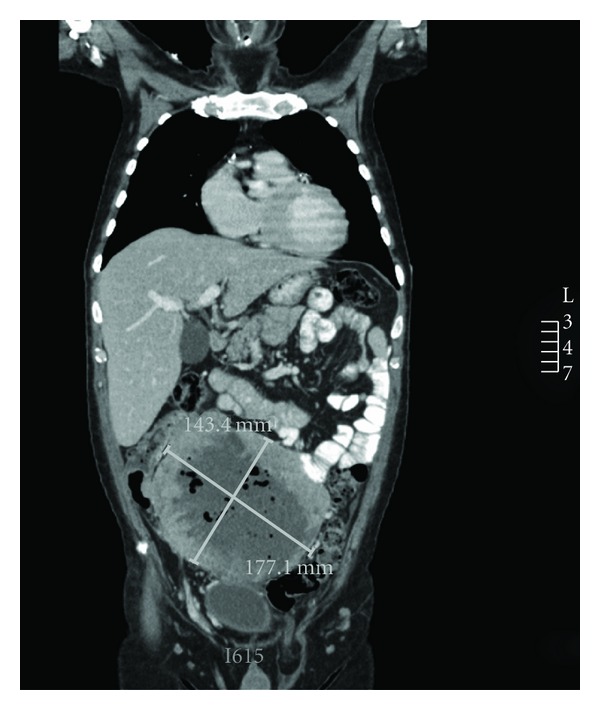
A representative cross-section of the mass on CT scan. The mass was secondarily infected following CT guided biopsy as indicated by the locules of gas within the tumour mass.

A 52-year-old man presented with iron-deficiency anaemia (haemoglobin of 58 g/L) and 40 kg weight loss for investigation. He had previously undergone colonoscopy for family history of colorectal cancer 2 years prior to presentation with no significant findings reported. Physical examination revealed a large mildly tender mass in the right lower quadrant of his abdomen. According to the patient, this mass had been present and slowly enlarging over two years. There was no generalised lymphadenopathy, and rectal examination was normal. Tumour markers (CEA, CA19.9) were normal. CT scan revealed a large necrotic mass measuring 19 × 17 cm (Figure 1) with no evidence of intra-abdominal or systemic dissemination. Two attempts at CT-guided biopsy did not yield sufficient material for diagnosis and was complicated by secondary infection of the necrotic tumour resulting in sepsis. He subsequently underwent exploratory laparotomy. At the time of surgery, the tumour seemed to have arisen from the ileocaecal region with direct invasion of an adjacent loop of jejunum. Excision of the mass with en bloc right hemicolectomy and small bowel resection was performed with two primary anastomoses. The patient made an uneventful recovery and was discharged two weeks postoperatively. 

**Figure 2:**
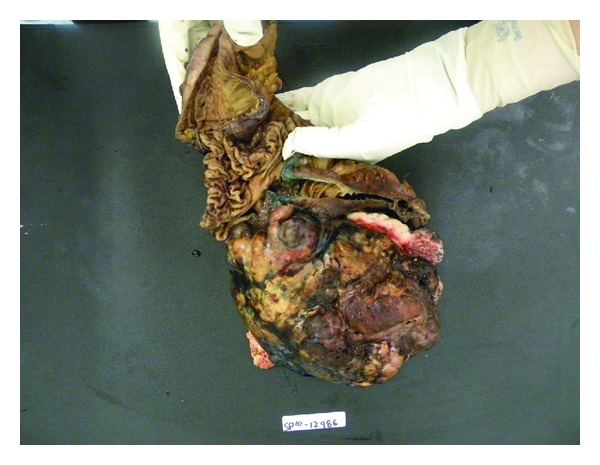
En bloc hemicolectomy specimen, showing cecum and terminal ileum with overlying tumour. Arrow points to part of the appendix wall.

**Figure 3:**
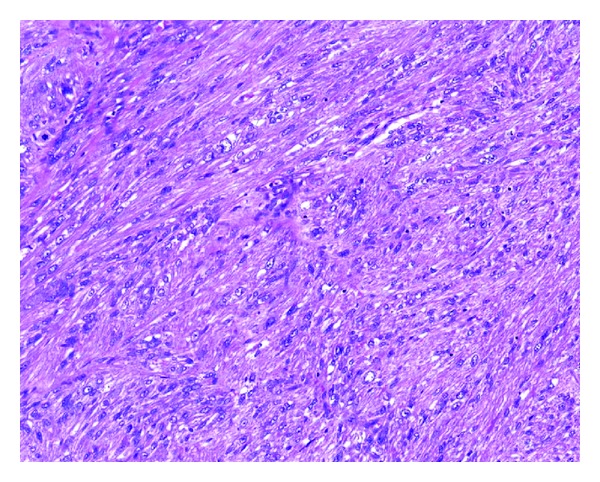
Haematoxylin and eosin section of the tumour under low power field, showing the highly cellular tumour with areas demonstrating smooth muscle differentiation.

Pathologic examination showed a large tumour arising from the wall of the appendix (Figure 2). Microscopically, the tumour is a malignant pleomorphic tumour comprising spindle cells, epithelioid cells, giant cells, and myomatous areas (Figure 3). The tumour stained strongly for SMA and Caldesmon, weakly for desmin and calponin, and was negative for CD 117, CD 34, S100, Melan A, CD 10, and CD 68. This staining pattern was consistent with a dedifferentiating pleomorphic leiomyosarcoma [1]. The tumour was highly cellular with up to 16 mitoses per high power field and variable areas of necrosis. Surgical margin appeared to be involved anteriorly despite wide excision incorporating the posterior rectus sheath and rectus muscle. All other margins were otherwise clear microscopically. None of 16 lymph nodes examined were involved with tumour.

An outpatient PET scan did not demonstrate any residual intra-abdominal or distant disease. Radiation and medical oncology reviews were sought but in view of complete surgical excision and nature of leiomyosarcomas, and the patient initially was not offered any adjuvant therapy. Unfortunately, the patient represented with symptomatic anaemia nine months later and was found to have local and systemic recurrence on CT and PET scan. He is currently receiving radiotherapy for symptomatic control.

## 2. Discussion

Appendiceal neoplasms are rare and occur in approximately 0.9–1.4% of all appendicectomies [2]. Carcinoid tumours and adenocarcinomas comprise the majority of these tumours with reported incidences of 32–85% and 36–65%, respectively [2, 3]. Much less commonly, lymphomas and sarcomas can also arise within the appendix with respective incidences of 1.7% and <1% [3]. In a 10-year single institutional review by O'Donnell et al., 22 cases of malignant appendiceal tumours were identified from 2154 (1.02%) appendicectomy specimens [2]. Of these tumours, the majority were carcinoids and adenocarcinomas with no reported cases of sarcomas. In a large 32-year review of the SEER (surveillance, epidemiology, and end results registry) database, sarcomas accounted for only 6 of 2791 (0.21%) cases of malignant appendiceal tumours, making it an extremely rare appendiceal malignancy [3]. Further, all 6 reported cases were Kaposi sarcomas with no leiomyosarcomas [3]. To date, the largest body of work specific to appendiceal leiomyosarcoma can be attributed to Hatch et al. and Charache with a total of 5 reported cases of appendiceal leiomyosarcoma, further testifying to the rarity of the tumour [4, 5]. Since the latest review by Hatch et al. in 2000, only two other cases have been identified, including the current report [6].

Due to its rarity, little is known specifically about appendiceal leiomyosarcomas. Instead, most data is extrapolated from the behavior of sarcomas and experience from treatment of leiomyosarcomas of the colon. Based on the review by Hatch et al., most leiomyosarcoma of the appendix and colon occur between the age of 20 and 70 and that most are symptomatic, presenting with pain, acute or chronic gastrointestinal bleeding, weight loss, or constipation [4]. Preoperative diagnosis is uncommon which is not surprising given the rarity of the tumour. However, it is also noteworthy that despite preoperative biopsies, diagnosis often remains elusive until definitive surgical resection due to difficulty with smooth muscle identification under light microscopy, as exemplified by this case [4]. In the current case, the tumour was large measuring 19 × 17 × 12 cm. Of all previously reported cases, only one reported the size of the tumour, therefore, making difficult to compare the sizes of tumours at presentation [7]. 

As with the management of any soft-tissue sarcoma, surgical excision with en bloc excision of contiguously affected viscera with clear resection margins is the mainstay of treatment and the only option that offers a chance at cure [3, 4]. Unlike adenocarcinomas, whereby lymphatic spread is the commonest mode of dissemination, there is no role for extended lymphadenectomy in leiomyosarcomas. As exemplified by this case, none of the 16 lymph nodes examined were involved with tumour. Adjuvant radiotherapy and chemotherapy have shown to have little effect on disease progression and outcome [4]. Response rates to anthracycline-based chemotherapy are low (15–30%) with no significant improvement in overall survival [4]. Prognostic factors include number of mitoses and extent of disease at presentation [4, 7]. And as exemplified by this case, the size of the tumour does not correlate with likelihood of metastases. More data and research are required to better understand and guide management of this rare tumour.
